# Occurrence and the ecological implication of a tropical anguillid eel *Anguilla marmorata* from peninsular Malaysia

**DOI:** 10.3897/zookeys.695.13298

**Published:** 2017-09-05

**Authors:** Siti Raudah Abdul Kadir, Mohamad Hafiz Farhan Abdul Rasid, Kok Onn Kwong, Li Lian Wong, Takaomi Arai

**Affiliations:** 1 Institute of Oceanography and Environment, Universiti Malaysia Terengganu, 21030 Kuala Terengganu, Terengganu, Malaysia; 2 Institute of Tropical Aquaculture, Universiti Malaysia Terengganu, 21030 Kuala Terengganu, Terengganu, Malaysia; 3 School of Biological Sciences, Universiti Sains Malaysia, Minden, Penang, Malaysia; 4 Environmental and Life Sciences Programme, Faculty of Science, Universiti Brunei Darussalam, Jalan Tungku Link, Gadong, BE 1410, Brunei Darussalam

**Keywords:** Anguillid eel, giant mottled eel, molecular, species identification, tropical fish

## Abstract

Recent studies suggested that accurate species identification in the tropical anguillid eels based on morphological examination requires confirmation by molecular genetic analysis. Previous studies found that two tropical anguillid eels, *Anguilla bicolor bicolor* and *A. bengalensis bengalensis*, were found in peninsular Malaysia (West Malaysia) based on morphological and molecular genetic analyses. This study is the first record of *A. marmorata* in peninsular Malaysia confirmed by both morphological and molecular genetic analyses. The present study also suggests that accurate tropical eel species identification is difficult by morphological identification alone; therefore, molecular genetic analysis is needed for precise species confirmation.

## Introduction

The anguillid eels of the genus *Anguilla* Schrank are widely distributed throughout the world. These eels have a catadromous life history, migrating between inland or coastal growth habitats and offshore spawning locations. Nineteen species or subspecies of *Anguilla* have been reported worldwide, thirteen of which occur in tropical regions. Of the thirteen species/subspecies found in tropical areas, seven species/subspecies inhabit western Pacific around Indonesia and Malaysia: *A. celebesensis* Kaup, *A. interioris* Whitely, *A. bengalensis bengalensis* Gray, *A. marmorata* Quoy & Gaimard, *A. borneensis* Popta, *A. bicolor bicolor* McClelland and *A. bicolor pacifica* Schmidt (Ege 1939, Castle and Williamson 1974, Arai et al. 1999).

Molecular phylogenetic research on freshwater eels have revealed that tropical eels are the most basal species originating in the Indonesian and Malaysian regions and that freshwater eels radiated from the tropics to colonize temperate regions (Minegishi et al. 2005). Recently, freshwater eel biology such as species composition, distribution, and life history has gradually accumulated in tropical eel species in Malaysian waters (e.g., Arai et al. 2012, 2015, 2017a, b, Arai and Chino 2013, Arai 2014, Abdul Kadir et al. 2015, Arai and Wong 2016, Wong et al. 2017). Identification of eels at the species level using morphological examination only is difficult because of similarities and overlapping of morphological characters, particularly in tropical anguillids (Arai et al. 2015, Arai and Wong 2016). Currently, two anguillid eels, *Anguilla bengalensis bengalensis* and *A. bicolor bicolor*, have been confirmed to occur in peninsular Malaysia (Arai et al. 2015, Arai and Wong 2016).

In the present study, two anguillid specimens were collected and examined from the Pondok Upeh River, Penang Island. As Arai et al. (2015) and Arai and Wong (2016) suggested that tropical eel species identification could be accurately validated by molecular genetic analysis after morphological observation, the specimens were subjected to identification using both morphological and mitochondrial cytochrome oxidase subunit I (COI) 16S ribosomal RNA (16S rRNA) sequence analyses. This paper describes the first confirmed record of a tropical anguillid eel, *Anguilla marmorata*, from peninsular Malaysia.

## Materials and methods

### Eel samples and morphological analysis

**Figure 1.**
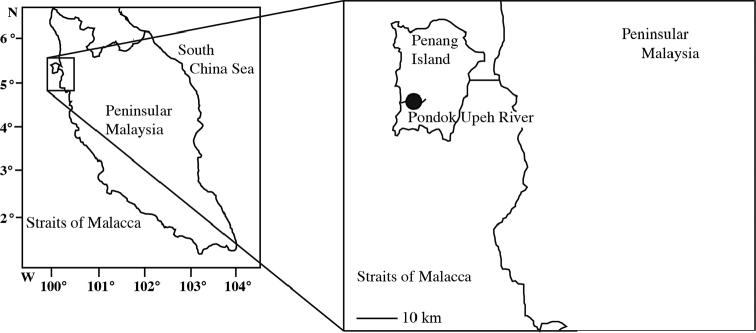
Sampling site in the Pondok Upeh River in Penang Island of peninsular Malaysia.

Two anguillid specimens were collected by hook and line by local people in the Pondok Upeh River in Penang Island of peninsular Malaysia on 2 April 2015 (Fig. 1).

External measurements follow Ege (1939) and Watanabe et al. (2004), and the data are shown in Table 1. The fin difference index (**FDI**), which is the distance between the verticals from the origin of the dorsal fin (**Z**) to the anus (ano-dorsal length) relative to the total length (***L*_T_**) (Ege 1939) was calculated as follows: FDI = 100 Z *L*_T_^-1^. The number of teeth in the mid part of the maxillary band is abbreviated as NMM.

**Table 1.** Morphometric characteristics of *Anguilla marmorata* and *A. bengalensis bengalensis* as revealed by molecular genetic analyses.

Specimen number	SP26	TB316
Species by molecular genetic	*A. marmorata*	*A. bengalensis bengalensis*
Species by morphology	*A. celebesensis*	*A. marmorata*
**Measurements (mm)**		
Total length (*L*t)	904	889
Standard length (SL)	886	975
Body weight (g)	2335	1579
Head length (HL)	136	127
Predorsal length (PD)	303	259
Preanal length (PA)	403	395
Length of intermaxillary-vomerine band (LV)	28.7	24.9
Length of left maxillary band (LM)	30.9	32.1
Number of teeth of mid part of maxillary band (NMM)	5	1
Width of mid part of maxillary band (WMM)	4.4	1.1
FDI (%)	11	15

### Molecular genetic analysis

Two mitochondrial genes, cytochrome oxidase *c* subunit 1 (COI) and 16S ribosomal RNA (16S rRNA), were used. DNA was extracted from a dorsal fin clip of each specimen using Gentra Puregene Tissue Kit (QIAGEN, USA), according to the manufacturer’s instructions. Total DNA concentration and quality was quantified using BioPhotometer Plus spectrophotometer (Eppendorf, Germany). Both mitochondrial cytochrome COI and 16S rRNA genes were amplified using primer pairs (Table 2) to validate the species identity of each specimen. PCR reaction and condition for COI gene was performed according to Abdul Kadir et al. (2015), whereas PCR amplification for 16S rRNA gene was conducted according to Arai and Wong (2016). PCR amplicons were purified using QIAquick® PCR Purification Kit (QIAGEN, USA), labeled with BigDye Terminator v.3.1 Cycle Sequencing Kit (Applied Biosystems Inc., USA), and sequenced bi-directionally on an ABI PRISM 3730xl Genetic Analyzer. Generated sequence trace files were manually edited and assembled using SeqMan Pro application in DNASTAR version 6.0 (DNASTAR Inc., USA). The contig sequences were compared for percentage similarity with the reference sequences in the GenBank using BLAST search. The sequences for both specimens were deposited to GenBank with accession numbers as listed in Table 3.

**Table 2.** Primers used in this study.

Gene	Primer Sequences	Sources
Cytochrome oxidase subunit I (COI)	**FF2d**: 5’TTCTCCACCAACCACAARGAYATYGG3’ **FR1d**: 3’CACCTCAGGGTGTCCGAARAAYCARAA5’	Ivanova et al. (2007)
16S rRNA	**L2510**: 5′CGC CTG TTT ATC AAA AAC AT 3’ **H3080**: 5′ CCG GTC TGA ACT CAG ATC ACG T 3′	Palumbi et al. (1991)

## Results

**Figure 2.**
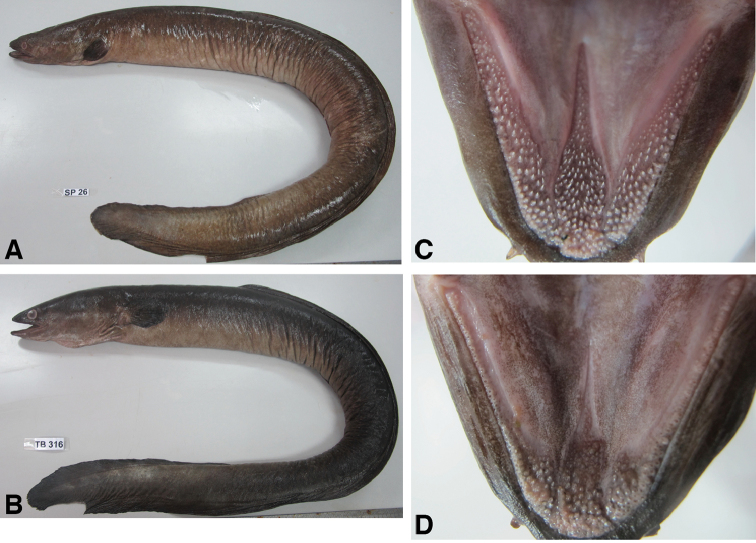
*Anguilla marmorata* and *A. bengalensis bengalensis* collected in the Pondok Upeh River in Penang Island of Peninsular Malaysia. **A** *Anguilla marmorata* (904 mm in TL) **B** *Anguilla bengalensis bengalensis* (889 mm in TL) **C** Wide maxillary bands of teeth of *A. marmorata* **D** Narrow maxillary bands of teeth of *A. bengalensis bengalensis*. DNA was extracted from dorsal fin clip of each specimen, and hence the posterior dorsal fin of each specimen is lacking.

The two specimens examined in this study had variegated markings on the body (Fig. 2a, b). However, one (SP26) had wide maxillary bands of teeth with a small number of NMM teeth (Fig. 2c) and 11 of FDI while the second specimen (TB316) had narrow maxillary bands of teeth with a higher NMM (Fig. 2d) and 15 of FDI.

SP26 was assigned into the first species group of the genus *Anguilla* (*A. celebesensis*, *A. interioris*, *A. megastoma* Kaup, *A. luzonensis* Watanabe, Aoyama and Tsukamoto) based on the variegated skin and wide maxillary bands of teeth (Ege 1939, Watanabe et al. 2004, Arai and Wong 2016). TB316 was assigned to the second group (*A. bengalensis bengalensis*, *A. bengalensis labiata* Peters, *A. marmorata*, *A. reinhardtii* Steindachner) based on variegated skin and narrow maxillary bands of teeth (Ege 1939, Watanabe et al. 2004, Arai and Wong 2016).

The geographical distribution of anguillids is used in combination with key morphological characteristics to determine the classification of each species into four groups. Within the first group, *A. interioris*, *A. megastoma*, *A. luzonensis* exist in New Guinea, Solomon Islands, New Caledonia, Fiji Islands, Cook Islands, and northern Philippines (Ege 1939). Therefore, SP26 was considered to be *A. celebesensis* with a range of FDI from 6 to 12 and of NMM from 4 to 9 (Ege 1939, Watanabe et al. 2004).

Within the second group, *A. bengalensis labiata* and *A. reinhardtii* exist in the mid-southeastern region of Africa and eastern Australia and Tasmania, respectively (Ege 1939). Therefore, both species were not considered when identifying the samples in the present study. The FDI of the other two species, *A. bengalensis bengalensis* and *A. marmorata*, was studied further. According to the key morphological characteristics used for their identification (Ege 1939, Watanabe et al. 2004), the FDI of *A. marmorata* is in the range of 12 to 20, higher than that of *A. bengalensis bengalensis*, which ranges from 8 to 14 (Ege 1939, Watanabe et al. 2004). Based on the FDI of TB316, which equals 15, this specimen was identified as *A. marmorata*.

A total contrast was found in the species identification outcomes in the molecular genetic analysis when compared to the morphological observation. As shown in Table 3, molecular identification based on two genes confirms that SP26, which was morphologically identified as *A. celebesensis*, was in fact *A. marmorata*. On the other hand, specimen TB316 identified as *A. marmorata* was actually confirmed as *A. bengalensis bengalensis* based on the genetic results.

**Table 3.** Validation of species identity of collected specimens based on mitochondrial cytochrome oxidase subunit I (COI) and 16S ribosomal RNA (16S rRNA) genes using BLAST search in GenBank.

Morphological identification	Genetic identification	% Max identity (BLASTn)		GenBank Accession Number	
		COI	16S rRNA	COI	16S rRNA
SP26 *A. celebesensis*	*Anguilla marmorata*	94	99	KT728354	KT728352
TB316 *A. marmorata*	*Anguilla bengalensis bengalensis*	99	99	KT728353	KT728351

## Discussion

The findings from this and previous studies (Arai et al. 2015, Arai and Wong 2016) have suggested that tropical eel species identification could be accurately validated by molecular genetic analysis after morphological observation. Misidentification using morphology has been reported for *A. borneensis* and *A. bicolor bicolor* (Arai and Wong 2016). Likewise, same mismatches between morphological identification and genetic identification were also found in TB316. Although TB316 was identified as *A. marmorata* by morphological key characters, it was identified as *A. bengalensis bengalensis* by molecular genetic analyses. The inconclusive morphological identification of these specimens is not merely a technical error, but, rather, is due to the inadequacy of the description of the key of morphological characteristics. Further, the species identity of both specimens from this study have been validated by a concrete molecular data of previous studies, in which putative voucher specimens of *A. bengalensis bengalensis* (Arai and Wong 2016) and *A. marmorata* (Wong et al. 2017) were morphologically examined for species confirmation. This verification process is necessary to ensure that our molecular data is solely based on correctly identified species, instead of referring to sequences in GenBank which may likely derived from misidentified specimens.

In previous studies, *Anguilla marmorata* was reported to exist in Langkawi Island, peninsular Malaysia (Ahmad and Lim 2006, Azmir and Samat 2010). However, after a thorough morphological re-examination by Ahmad and Lim (2006) of one sample of *A. marmorata* preserved in formalin, Arai (2014) discovered that the true identify of that particular sample was *A. bengalensis bengalensis*. In fact, the difficulty in distinguishing between *A. marmorata* and *A. bengalensis bengalensis* is augmented by their overlapping morphological characteristics. Furthermore, recent molecular studies also found that all eels that possess skin with variegated markings were identified as *A. bengalensis bengalensis* (Arai et al. 2015, Arai and Wong 2016); however, this is the first description of the occurrence of *A. marmorata* in peninsular Malaysia identified by molecular genetic analyses. The present and previous studies all lead to the conclusion that currently three eels, i.e., *A. bengalensis bengalensis*, *A. marmorata*, and *A. bicolor bicolor*, occur in peninsular Malaysia.

According to Jespersen (1942), the anguillid eels distributed in Java and Sumatra may have their spawning areas situated off the south-western coast of Sumatra. *Anguilla marmorata* in Malaysia might originate from spawning areas off Sumatra. However, the distance between the spawning area and recruitment area in peninsular Malaysia is considerably larger than the distance between the islands of Java and Sumatra; therefore, the abundance of specimens that survive to reach peninsular Malaysia might be quite low. This would make *A. marmorata* difficult to identify in the area. Further field sampling should be undertaken, along with accurate species identification, in order to better understand the details of species composition and distribution of the tropical anguillid eels.
